# Evaluating risk factors for *Clostridiumdifficile* infection in adult and pediatric hematopoietic cell transplant recipients

**DOI:** 10.1186/s13756-015-0081-4

**Published:** 2015-10-14

**Authors:** Nicole M. Boyle, Amalia Magaret, Zach Stednick, Alex Morrison, Susan Butler-Wu, Danielle Zerr, Karin Rogers, Sara Podczervinski, Anqi Cheng, Anna Wald, Steven A. Pergam

**Affiliations:** Vaccine and Infectious Disease Division, Fred Hutchinson Cancer Research Center, Seattle, WA USA; Department of Biostatistics, University of Washington, Seattle, WA USA; Department of Laboratory Medicine, University of Washington, Seattle, WA USA; Seattle Children’s Hospital, Seattle, WA USA; Department of Pediatrics, University of Washington, Seattle, WA USA; Washington State Department of Health, Shoreline, WA USA; Department of Medicine, University of Washington, Seattle, WA USA; Department of Epidemiology, University of Washington, Seattle, WA USA; Infection Prevention, Seattle, WA USA

**Keywords:** *Clostridium difficile*, Hematopoietic, Transplant, Allogeneic, Pediatric, Adult

## Abstract

**Background:**

Although hematopoietic cell transplant (HCT) recipients are routinely exposed to classic risk factors for *Clostridium difficile* infection (CDI), few studies have assessed CDI risk in these high-risk patients, and data are especially lacking for pediatric HCT recipients. We aimed to determine incidence and risk factors for CDI in adult and pediatric allogeneic HCT recipients.

**Methods:**

CDI was defined as having diarrhea that tested positive for *C. difficile* via PCR, cytotoxin assay, or dual enzyme immunoassays. We included all patients who received an allogeneic HCT from 2008 to 2012 at the Fred Hutchinson Cancer Research Center; those <1 year old or with CDI within 8 weeks pre-HCT were excluded. Patients were categorized by transplanting hospital (“adult” or “pediatric”) and followed for 100 days post-HCT.

**Results:**

Of 1182 HCT recipients, CDI was diagnosed in 17 % (33/192) of pediatric recipients for an incidence of 20 per 10,000 patient-days, and 11 % (107/990) of adult recipients for an incidence of 12 per 10,000. Pediatric recipients were diagnosed a median of 51 days (interquartile range [IQR]: 5, 72) after HCT and adults at 16 days (IQR = 5, 49). Compared with calendar year 2008, pediatric recipients transplanted in 2012 were at increased risk for CDI (hazard ratio [HR] = 3.99, *p* =.02). Myeloablative conditioning increased CDI risk in adult recipients (HR = 1.81, *p* =.005).

**Conclusions:**

Pediatric and adult allogeneic recipients are at high risk of CDI post-HCT, particularly adult recipients of myeloablative conditioning. Differences in CDI incidence between children and adults may have resulted from exposure differences related to age; therefore, separately evaluating these groups should be considered in future CDI studies.

## Background

*Clostridium difficile* infection (CDI) is the most common cause of nosocomial infectious diarrhea in the US [1–5], resulting in substantial morbidity and mortality among hospitalized patients. Costs associated with this healthcare-associated pathogen, the emergence of hypervirulent strains of *C. difficile*, and reports of increasing incidence and severity of CDI in both hospitals and in the community have led to major efforts to control and prevent this infection [1–5].

Patients undergoing allogeneic hematopoietic cell transplantation (HCT) typically experience one or more classic CDI risk factors during their care, and have been shown to be at increased risk for CDI when compared to both general and other cancer patient populations [6]. However, incidence estimates among allogeneic HCT recipients have varied from 2–27 % [7–16]. Furthermore, the risk imposed by factors exclusive to allogeneic HCT recipients, such as acute graft-versus-host disease (GVHD) and conditioning regimens, differ across studies [8–10, 15, 17]. It is unclear if differences in center-based transplant care, patient populations, or institutional testing strategies contribute to this variance.

In addition, studies on CDI among allogeneic HCT recipients have focused exclusively on adults or adult-dominated cohorts, with minimal data presented exclusively on CDI among pediatric HCT recipients. In the general population, the burden of CDI in pediatric patients is increasing, and the associated morbidity and mortality often differs from adults [18–20]. Even among pediatric patients, the risk of CDI varies by age: younger children have a higher rate of CDI [19, 21]; infants have a high risk of asymptomatic carriage of *C. difficile* and may serve as a reservoir for infection [22–25]. CDI data in pediatric HCT recipients have not been well described.

Given the variance in results across studies and the need for additional pediatric data, we examined the incidence and risk factors of CDI among two cohorts of allogeneic HCT recipients at a large cancer center.

## Methods

### Study design and population

We extracted demographic and medical data from a prospectively collected database on pediatric and adult HCT recipients from 56 days (8 weeks) pre-HCT through 100 days post-HCT. Diagnosis of CDI and other post-transplant outcome data were identified through the electronic database and confirmed with subsequent chart review. Study activities were approved by the Fred Hutchinson Cancer Research Center (FHCRC) Institutional Review Board, and participants provided written informed consent to collect and analyze data for research purposes according the principles of the Declaration of Helsinki.

All patients who received an allogeneic HCT at the FHCRC in Seattle, WA between January 1, 2008–December 31, 2012 were eligible for inclusion in this study. Patients with preexisting CDI, defined as a positive test for *C. difficile* toxin A or B or genes *tcdA* or *tcdB* within 8 weeks prior to transplantation [26], were excluded from analysis to avoid misclassification of recurrent CDI events as incident cases. In addition, infants under one year of age were excluded.

### Transplant standards, antimicrobial prophylaxis, and surveillance

Patients received acyclovir or valacyclovir prophylaxis for prevention of herpes simplex viruses and varicella-zoster virus; cord blood transplant recipients after June 2008 received high-dose valacyclovir for cytomegalovirus (CMV) prevention [27]. Most patients received daily fluconazole as antifungal prophylaxis; those with known or presumptive fungal infections received either voriconazole, posaconazole or liposomal amphotericin B. All patients received standard *Pneumocystis jirovecii* prophylaxis with trimethoprim-sulfamethaxazole, dapsone, or atovaquone following engraftment. Screening and preemptive therapy for CMV have been described elsewhere [27, 28]. Conditioning for HCT, as well as prophylaxis and treatment of GVHD, were performed using current standardized protocols [29]. Acute GVHD was graded according to standard criteria [30].

All adult patients who developed neutropenia (absolute neutrophil count <500/ *μ*l) received levofloxacin prophylaxis until neutrophil recovery. All pediatric patients under the age of 18 and adults with allergy/intolerance to levofloxacin received ceftazidime for neutropenic prophylaxis. Adult patients who developed neutropenic fever underwent routine blood culture testing and adults preferentially received ceftazidime as first line therapy, whereas children received either cefepime or a carbapenem. Gram-positive coverage with vancomycin was generally only used for patients with severe mucositis or those with documented or suspected gram positive infections. Decisions regarding continuation of therapy, changes in antibiotic coverage, and clinical and laboratory assessment of fever were made by the primary care team.

### Definitions

#### Transplanting hospital

Adults patients were hospitalized at the University of Washington Medical Center (UWMC). Children (<18 years) and few young adults (≥18 years) received inpatient care at Seattle Children’s Hospital (SCH). Both adults and children had outpatient care at the Seattle Cancer Care Alliance ambulatory clinic. All analyses were conducted separately by the site of transplant. For brevity, patients transplanted at the pediatric hospital will heretofore be referred to as ‘pediatric(s)’ or ‘children’ and those at the adult hospital as ‘adult(s).’

#### *C. difficile* testing

*C. difficile* testing was ordered at the discretion of the primary team when patients developed new onset diarrhea. Per national guidelines, *C. difficile* testing was performed only on liquid stool [31]. Repeat testing following a positive test (“test-of-cure”) was actively discouraged in adult transplants, but was often performed in inpatient pediatric transplants for removal from inpatient isolation.

During the study observation period, various testing methods were employed throughout the FHCRC network. Adult transplants: Before 2010, *C. difficile* testing was performed using EIA (enzyme immunoassay) tests for both *C. difficile* GDH (glutamate dehydrogenase antigen) (C. Diff Quik Chek by TechLab; Blackburg, VA) and *C. difficile* toxins A and B (Toxin A/B Quik Chek by TechLab, Blackburg, VA). Any specimens testing GDH positive and toxin negative were subjected to additional testing with a real-time PCR test for the *tcdB* gene (laboratory-developed test). CTA (cytotoxin assay) for toxin B detection was also conducted at physician discretion [32]. Beginning in 2010, EIA and CTA testing was replaced center-wide with a standalone PCR test for the *tcdB* gene (Xpert *C. difficile* by Cepheid, Sunnyvale, CA). Pediatric transplants: *C. difficile* testing included a combined test for GDH and toxins A and B (C. Diff Quik Chek Complete by TechLab, Blackburg, VA); if found GDH positive and toxin negative, a real-time PCR test for toxins A and B (laboratory-developed) was conducted.

**Fig. 1:**
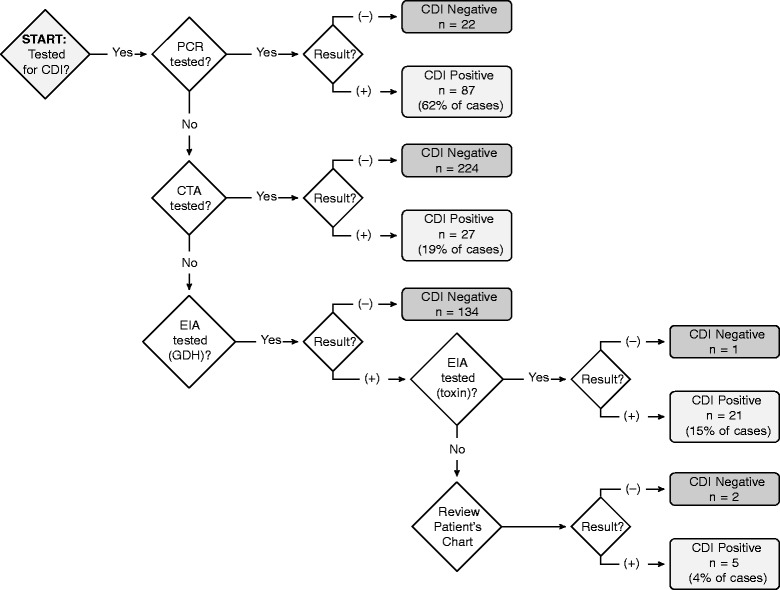
Algorithm to determine *C. difficile* infection (CDI) positivity for retrospective analyses, adult and pediatric populations combined. Definitions: PCR (polymerase chain reaction) for detection of gene *tcdB*, or both genes *tcdA* and *tcdB*; EIA (enzyme immunoassay) for detection of *C. difficile* GDA (glutamate dehydrogenase antigen); EIA for detection of toxins A & B; CTA for toxin B (cytotoxicity assay); review of patient’s chart was performed in order to manually review laboratory results and discharge/interim records

For the purposes of this study, CDI was defined as a single instance of liquid stool found positive for *C. difficile* toxins A or B, *tcdA* gene, or *tcdB* gene. Figure 1 depicts the algorithm used for both adult and pediatric transplant populations to determine CDI in this study.

### Statistical analyses

The association between potential risk factors (age, year of transplant, stem-cell source, conditioning regimen, and GVHD prophylactic regimen) and CDI risk were analyzed through both univariate and backward-eliminated (variable inclusion threshold of *p*<0.1) cause-specific multivariate Cox proportional hazards models, where observation time was censored at 100 days, death, re-transplant, or lost to follow-up. Additionally, onset of overall GVHD (grades ≥ II), GI GVHD (grades ≥ II), and inpatient acquisition (i.e. exposure to inpatient stay 3 days prior) were analyzed in a similar manner and were modeled as time-dependent variables. Multilevel indicator variables were first tested for global significance, and if found significant, individual levels were tested against the reference group for significance. Cumulative incidence functions were derived from the subdistribution hazard functions and plotted for each transplanting hospital (pediatric/adult), where death was treated as a competing risk. Yearly proportions of patients tested and diagnosed with *C. difficile* via any method (EIA/CTA/PCR) versus PCR were calculated. Statistical significance was defined at *p*≤.05. Data analyses were conducted using Stata version 13.1 (StataCorp; College Station, TX).

## Results and discussion

A total of 1240 allogeneic HCT recipients transplanted between 2008 through 2012 were eligible for inclusion in this study. Due to pre-transplant CDI, 58 patients (11 pediatrics and 47 adults) were excluded from primary analyses. Therefore, 1182 patients comprised the final study population, including 192 pediatric and 990 adult HCT recipients (Table 1). Pediatric and adult HCT recipients differed by graft type, as bone marrow (53 %) and cord blood (28 %) were the most common graft types transplanted among children, and peripheral blood stem cells (PBSC) were most commonly transplanted among adults (75 %). Myeloablative conditioning was significantly more frequent in children (91 %) than in adults (57 %, *p*<.0001).

**Table 1:** Patient demographics, by transplanting hospital

	Pediatric transplants	Adult transplants
Characteristic	(*n* =192)	(*n* =990)
Age (yr) – median (IQR)	11 (6, 15)	52 (42, 61)
Sex: male – n (%)	104 (54)	583 (59)
Race/Ethnicity – n (%)		
Caucasian	101 (53)	761 (77)
Hispanic	31 (17)	27 (3)
Asian Pacific-Islander	19 (10)	59 (6)
Black	6 (3)	17 (2)
Native American	2 (1)	8 (1)
Other	22 (12)	53 (5)
Unknown	11 (6)	65 (7)
Underlying Disease – n (%)		
ALL	67 (35)	107 (11)
AML	34 (18)	341 (34)
MDS	12 (6)	206 (21)
NHL	7 (4)	107 (11)
CML	6 (3)	36 (4)
HD	1 (1)	30 (3)
Other	65 (34)	163 (17)
Stem cell source – n (%)		
Bone marrow	102 (53)	155 (16)
PBSC	36 (19)	743 (75)
Cord blood	54 (28)	92 (9)
Donor – n (%)		
Sibling	52 (27)	300 (30)
Unrelated	80 (42)	523 (53)
Cord	54 (28)	92 (9)
Haploidentical	6 (3)	68 (7)
Conditioning regimen – n (%)		
Myeloablative	175 (91)	559 (57)
Nonmyeloablative	17 (9)	431 (44)
GVHD prophylaxis – n (%)		
CSP or FK506, with MTX	106 (55)	360 (36)
MMF regimen	74 (39)	539 (54)
Other/none	12 (6)	91 (9)
Inpatient days^a^ – median (IQR)	41 (31, 55)	18 (5, 28)

*Abbreviations*: *IQR* (interquartile range), *ALL* (acute lymphoblastic leukemia), *AML* (acute myeloid leukemia), *MDS* (myelodysplastic syndrome), *NHL* (non-Hodgkin lymphoma), *CML* (chronic myeloid leukemia), *HD* (Hodgkin’s lymphoma), *PBSC* (peripheral blood stem cells), *GVHD* (graft-versus-host disease), *CSP* (cyclosporine), *FK506* (tacrolimus), *MTX* (methotrexate), *MMF* (mycophenolate mofetil)

^a^While under observation (i.e. prior to censorship)

**Fig. 2:**
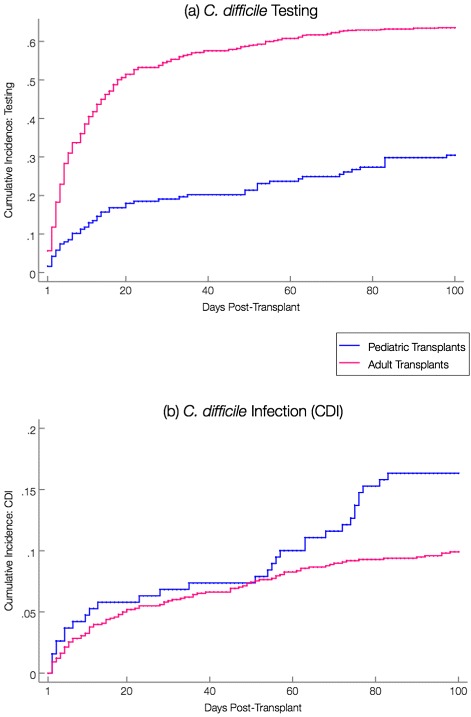
Comparison of 100-day post-allogeneic cumulative incidence curves between pediatric and adult transplants: **a** Incidence of *C. difficile* testing; **b** Incidence of CDI. Figure 2(a) displays the cumulative proportion of patients tested for *C. difficile*, and Fig. 2(b) displays cumulative incidence plots depicting the progression of *C. difficile* infection incidence over time. Note: Scales of graphs 2(a) and 2(b) differ

Thirty-three pediatric patients (17 %) developed a CDI episode at a median of 51 days (interquartile range [IQR] = 5, 72) after HCT for an incidence rate of 20 per 10,000 patient-days, compared with 107 adults (11 %) at a median of 16 days (IQR = 5, 49) for an incidence rate of 12 per 10,000 patient-days (Table 2). Figure 2 shows the cumulative proportion of patients tested for *C. difficile* (a) and the cumulative incidence of CDI (b).

**Table 2:** Summary statistics for *C. difficile* infection (CDI) within 100-days post-allogeneic transplant, per transplanting hospital

	Pediatric transplants	Adult transplants
Variable	(*n* =192)	(*n* =990)
Outcomes – n (%)		
CDI	33 (17)	107 (11)
Death	8 (4)	93 (9)
Lost to follow-up	0 (0)	1 (0)
Second transplant	0 (0)	11 (1)
No event	151 (79)	778 (79)
CDI incidence, per 10000 patient-days	20	12
CDI incidence, per transplant year – n (%)		
2008	4 (10)	17 (9)
2009	4 (10)	25 (12)
2010	5 (16)	23 (13)
2011	7 (17)	19 (9)
2012	13 (35)	23 (12)
Days to first CDI test – median (IQR)	22 (10, 58)	15 (5, 33)
Days to first positive test for CDI – median (IQR)	51 (5, 72)	16 (5, 49)
Positive tests / all tests (%)	33/125 (26.4)	107/1,308 (8.2)
Probability of testing while inpatient^a^	1.10 %	4.50 %
Probability of CDI while inpatient^a^	0.30 %	0.30 %

^a^Per inpatient day

**Fig. 3:**
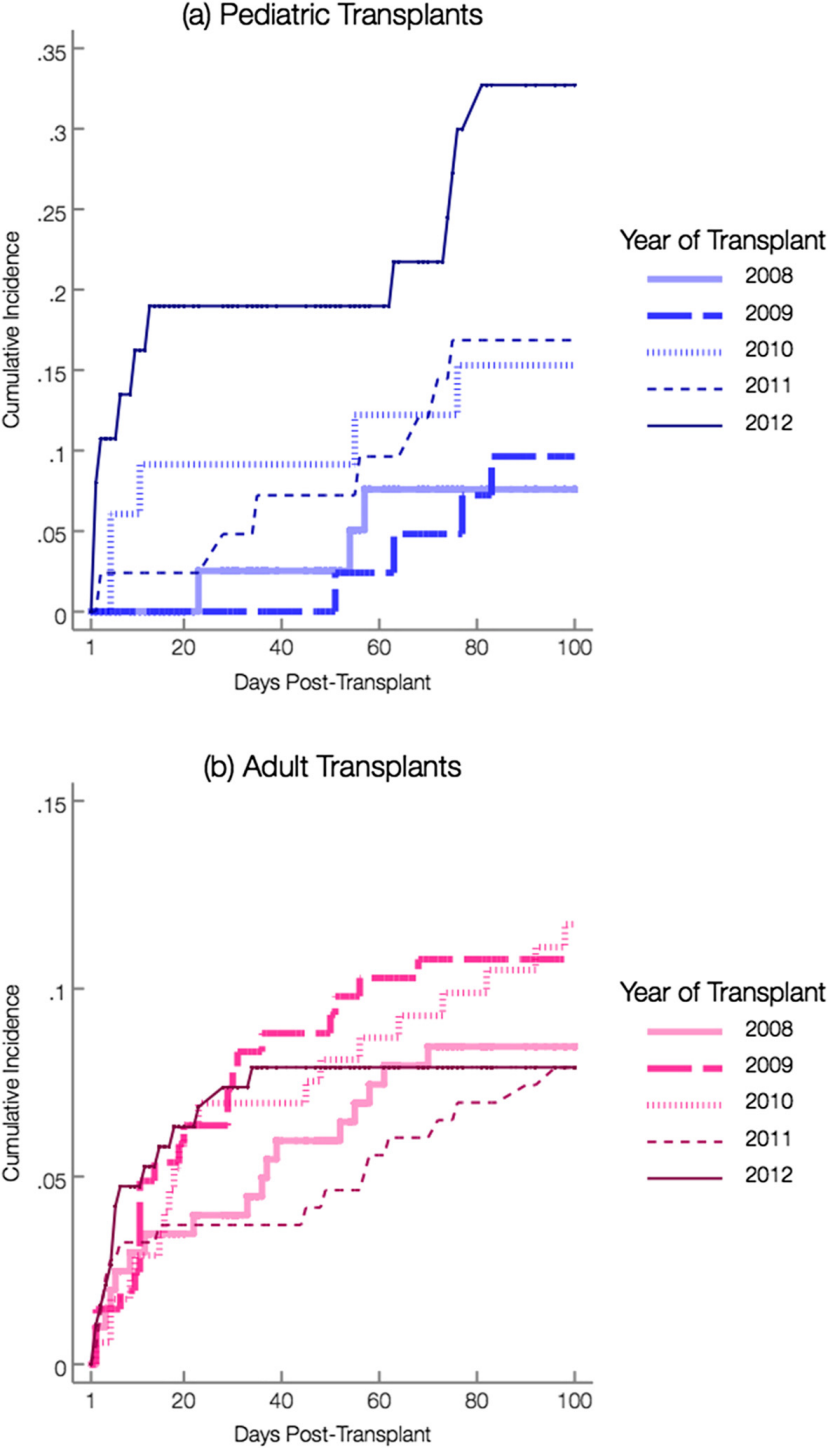
Comparison of yearly 100-day post-allogeneic cumulative incidence curves for CDI: **a** Pediatric Transplants; **b** Adult Transplants. Note: Scales of graphs 3(a) and 3(b) differ

**Fig. 4:**
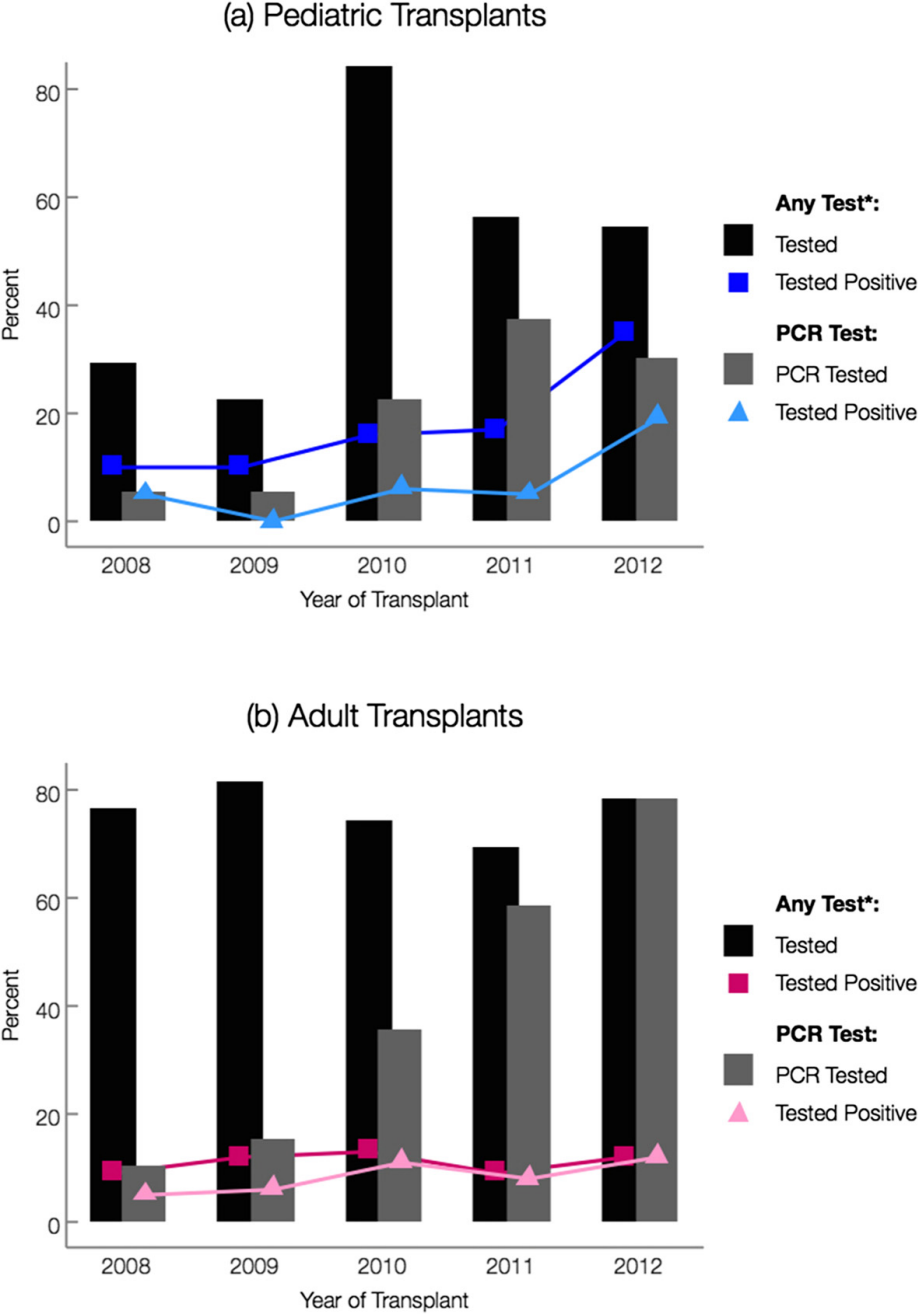
Proportion of pediatric patients tested positive per testing method (any vs. PCR) per year of transplant: **a** Pediatric Transplants; **b** Adult Transplants. *C. difficile* test includes any of the following tests: EIA (toxins A & B), CTA, or PCR (*tcdA* or *tcdB* gene). Dark bars indicated any *C. difficile* test, grey bars indicate those patients tested by PCR only. Colored lines (pink and blue) indicated percentage of positive results by testing type

**Fig. 5:**
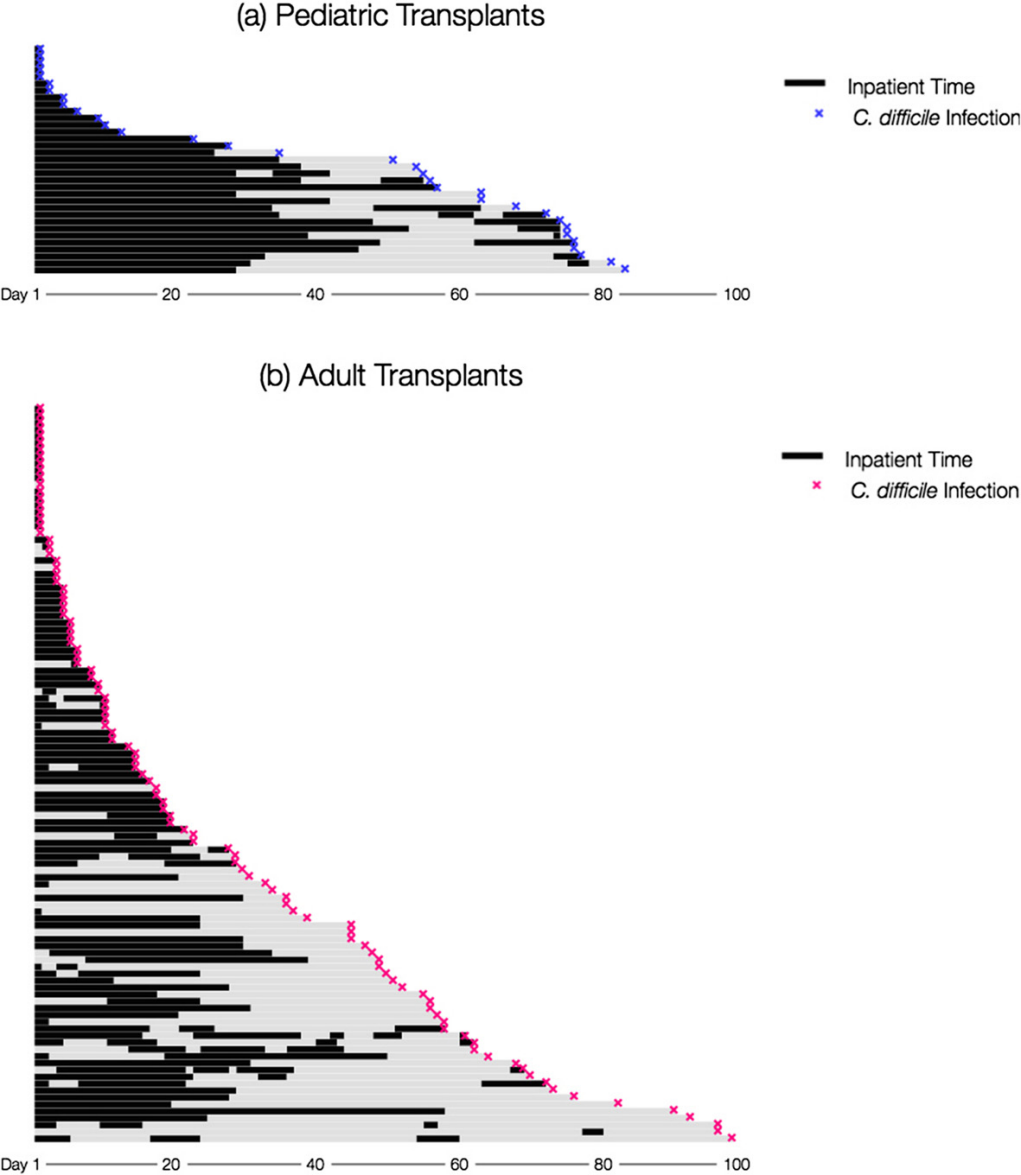
Inpatient time exposure after allogeneic HCT per each CDI case: **a** Pediatric Transplants, **b** Adult Transplants. Plotted longitudinal bars indicate inpatient and outpatient time over first 100 days until *C. difficile* event by type of transplant (5[a] = Pediatric, 5[b]= Adult) in patients with documented *C. difficile* infection

Yearly proportions of patients diagnosed with CDI were assessed for each site (Table 2; Fig. 3). Diarrhea leading to testing was common in both groups, but adults appeared to experience more testing for *C. difficile* (Fig. 4). Compared to 2008, pediatric HCT recipients experienced an increased risk of CDI in 2012 (HR = 4.0, *p* =.02). This increase appeared independent of yearly variations in PCR testing (Fig. 4). In contrast, none of the yearly cumulative incidences were significantly different from 2008 among adults, despite widespread testing in this group and a transition to standalone PCR testing in 2010 (Fig. 4). Among those diagnosed with CDI during our study, the median days to diagnosis suggested that adults were diagnosed earlier than children (16 days [IQR: 5, 49] vs. 51 days [IQR: 5, 72]; Table 2). Prior to diagnosis, all pediatric CDI cases were inpatient at some point during observation, contrary to adult CDI cases (Fig. 5).

In univariate analyses of the pediatric population, inpatient stay in the 3 days prior was significantly associated with an increase in CDI risk (HR = 1.8, *p* =.03; Table 3), although this variable was no longer significant in the multivariate model. In addition, year of transplant was a significant risk factor for pediatric CDI in both univariate and multivariate models, with year 2012 associated with 4 times the CDI risk of 2008 (HR = 4.0, *p* =.02; adjusted HR [aHR] = 4.0, *p* =.02). In univariate analyses of the adult patients, myeloablative transplantation significantly predicted CDI, while advanced age significantly decreased CDI risk. However, myeloablative transplant remained the sole significant risk factor for CDI in the multivariate model (HR = 1.8, *p* =.005; aHR = 1.8, *p* =.005).

**Table 3:** Univariate and multivariate Cox proportional hazards models for CDI post-allogeneic HCT, per transplanting hospital

	Pediatric transplants (*n* =192)				Adult transplants (*n* =990)			
	Univariate		Multivariate		Univariate		Multivariate	
Variable	HR	p	HR	p	HR	p	HR	p
Age group (yr)		.5						
1–5	Ref.		–		–		–	
6–10	0.8							
11–15	1.5							
16+	0.9							
<60	–				Ref.	.04	–	
60+					0.6			
Year of transplant		.03		.03		.42		
2008	Ref.		Ref.		Ref.			
2009	0.9		0.9		1.5		–	
2010	1.6		1.6		1.6			
2011	1.7		1.7		1			
2012	4		4		1.5			
Stem cell source		.32				.13		
Bone marrow	Ref.		–		Ref.		–	
PBSC	1.9				0.6			
Cord blood	1.2				0.8			
GVHD prophylaxis		.06				.13		
CSP or FK506, with MTX	Ref.		–		Ref.		–	
MMF regimen	0.9				0.7			
Other/none	3.1				1.1			
Conditioning regimen		.57				.005		.005
Nonmyeloablative	Ref.		–		Ref.		Ref.	
Myeloablative	1.5				1.8		1.8	
Overall GVHD ^*a*^		.24				.34		
Grades 0–I	Ref.		–		Ref.		–	
Grades II–IV	1.1				1.3			
Gut GVHD ^*a*^		.6				.83		
Grades 0–I	Ref.		–		Ref.		–	
Grades II–IV	0.6				0.9			
Inpatient acquisition ^*b*^	1.7	.02	–		2.1	.12	–	

*Abbreviations*: *PBSC* (peripheral blood stem cells), *GVHD* (graft-versus-host disease), *CSP* (cyclosporine), *FK506* (tacrolimus), *MTX* (methotrexate), *MMF* (mycophenolate mofetil), ^*a*^ *GVHD* onset modeled as time-dependent

^*b*^defined as inpatient stay within 3 days prior to the day of observation

Apparent differences in the timing and incidence of CDI between adult and pediatric recipients around day 50 post-HCT (Fig. 2) prompted an additional analysis that focused on the possibility of increased *C. difficile* acquisition among pediatric patients staying in a large, independently-owned outpatient housing facility. This facility was utilized by multiple pediatric families, including a large number of non-transplant patients. Although transplant patients resided in individual apartments in a separate section of the structure, other areas within the building (e.g. playrooms, common rooms) were available for use for anyone within the facility. Using admittance and discharge records, time contributed in the pediatric outpatient housing facility was determined. Location of CDI acquisition was defined as location (inpatient, outpatient facility housing, or outpatient elsewhere) of each CDI case 3 days pre-diagnosis [26]. Hazard estimates did not show a significant change in pediatric CDI acquisition risk from the outpatient housing facility compared to acquisition from other locations (outpatient facility housing vs. inpatient: HR = 0.67, p =.45; outpatient facility housing vs. outpatient elsewhere: HR = 2.16, *p* =.22). However, unlike other outpatient facilities, review of this facility’s infection control practices identified limited use of bleach in terminal room cleaning.

## Conclusions

This study of allogeneic HCT recipients demonstrated that children and adults are at high risk for CDI during the early (1–100 days) post-transplant period. Within this post-transplant timeframe, 11 % of adults and 17 % of children developed incident CDI. Diarrhea leading to *C. difficile* testing was common within the entire cohort but more widespread among adults. Among adult HCT recipients, only myeloablative conditioning was found to be associated with an increased risk for CDI. Our analyses detected year 2012 as the sole predictor for CDI among pediatric patients. In neither population did increases in PCR testing appear to influence the incidence of CDI.

Reported CDI incidence estimates in allogeneic HCT recipients vary between 2–27 % [7–16]. Variations in follow-up time, cohort size, the inclusion of autologous recipients or the inclusion of pre-transplant cases may have contributed to differences in reported incidence estimates. As HCT patients have demonstrated a high risk of CDI recurrence [12, 13], the inclusion of patients with preexisting CDI may also inflate incidence estimates. Furthermore, the routine application of PCR testing may contribute to higher rates of *C. difficile* [31, 33, 34]. While the 2010 transition to standalone PCR testing in our adult transplant patients did not appear to increase CDI incidence, this finding may have resulted from incorporation of PCR into the prior multistep testing protocols.

Variations in CDI incidence across studies may also reflect differences in pre-transplant asymptomatic *C. difficile* colonization. Diarrhea is extremely common in HCT and frequently leads to *C. difficile* testing, as seen in our study. With such frequent testing, asymptomatic *C. difficile* can be misidentified as true CDI [10, 15]. However, it is also true that patients with asymptomatic colonization can progress to symptomatic infection [10, 15, 17, 35]. Colonization rates could not be determined in our cohort, although the number of early CDI events suggests that pre-transplant colonization may have contributed to adult post-transplant CDI; the role of colonization in pediatric HCT is unknown and requires additional study.

Two prior analyses [8, 9] have linked GVHD (overall or GI) with CDI. However, the directionality of this association is unclear, as GVHD precedes CDI in some individuals and follows CDI in others [35]. One study accounting for this directionality found that overall/GI GVHD significantly predicted the development of CDI between 2 months to one year post-transplant [12]. Our results found that neither overall GVHD nor GI GVHD (grades ≥2) significantly predicted subsequent CDI. We may have seen similar associations had we observed our patients beyond 100 days.

Pediatric HCT recipients may experience increased opportunities for nosocomial transmission, as children in our center tend to spend more time inpatient than adults. Exposure to other children and fomites (e.g. toys) may facilitate *C. difficile* within pediatric facilities, particularly since asymptomatic colonized children may act as *C. difficile* reservoirs [22–25] and have previously been linked to transmission [36, 37]. Despite evidence suggesting CDI risk is unique in pediatrics, many published studies do not discern between pediatric and adult HCT recipients. Smaller retrospective studies of pediatric allogeneic HCT recipients have reported lower incidence estimates (9 % [38] and 13 % [11]) than the 17 % observed in our study. One prospective study performed weekly surveillance cultures and found a similar incidence to our study, with 16 % of pediatric allogeneic recipients found positive (irrespective of stool consistency) [39].

None of the HCT-associated risk factors evaluated in our study predicted CDI in children. Although calendar year 2012 did predict increased pediatric CDI incidence, pediatric testing practices were consistent during the study, suggesting that other unmeasured factors may be responsible for such changes. When late post-transplant incidence in pediatric HCT recipients prompted an evaluation of CDI acquisition at an independent outpatient facility, our analysis found no statistical associations. However, we did identify areas of improvement, most notably the addition of bleach to terminal cleaning at this facility. These findings provide a reminder to assure best practices for infection control in outpatient residential facilities and care centers geared toward pediatric populations [40].

Unfortunately, we were limited in our ability assess frequency of outpatient visits, or the duration and type of antibiotic use. Therefore, incidence and risk differences between pediatric and adult patients were not tested for statistical significance due to the presence of these potential confounders. Although ours is the largest study to address CDI in pediatric transplant patients, the limited size of our pediatric population still restricted our ability to detect associations of smaller magnitudes. Furthermore, as is typical with studies of HCT patients, many aspects of the transplant experience are not independent of one another, allowing for opportunities for possible co-linearity during multivariate risk factor analyses.

In conclusion, our study provides data on CDI incidence and risk factors among both pediatric and adult patients after allogeneic HCT. While our study provides further confirmation that both populations are at high risk of CDI during the first 100 days post-transplant, the implementation of standalone PCR testing did not appear to increase the rate of CDI diagnosis at our center. Differences in CDI risk between adults and children, as previously observed in non-transplant populations and suggested by our study, support the differentiation of adults from children when assessing post-transplant CDI. Due to small numbers of pediatric HCT recipients at most centers, future multicenter studies assessing CDI incidence in this population are needed.
